# The Coming of a Unified County Medical Service, and How It Will Affect the Voluntary Hospital

**Published:** 1910-10-29

**Authors:** Sidney Webb


### SPECIAL INSTITUTIONAL ARTICLE.

### The Coming of a Unified County Medical Service, and how it will Affect the Voluntary Hospital.

By MRS. SIDNEY WEBB, D.Litt.

(Abstract of a Paper read before the British Hospitals Association.)

The Royal Commission on the Poor Law brought out very forcibly three facts, which, whatever may be our prepossessions or opinions, are likely to be of dynamic potency. Firstly, that our Poor Law and our pauperism (on which we are this year spending out of the rates and taxes in the United Kingdom close upon twenty millions sterling) is, to the extent of at least one-third, and perhaps one-half, dealing with the destitution of people stricken with sickness—the greater part of it patently neglected sickness, sadly premature sickness, plainly postponable sickness; in a word, preventable sickness, which we are to-day not preventing. Secondly, that in our public arrangements for dealing with sickness we have got into a muddle, far greater than is commonly realised, of duplicated services, rival authorities, overlapping of work, waste of public money, and confusion of principles —maintaining on the one hand an ubiquitous rate-supported Poor-Law medical service, with four thousand badly-paid Poor-Law doctors, *forbidden to treat sickness unless and until it is complicated by destitution*, and then charged to give "medical relief "(!); on the other hand, an equally ubiquitous rate-supported Public Health medical service, with several thousand doctors, nurses, health visitors, etc., now dealing largely with the very same diseases as the Poor-Law medical service, but on a diametrically opposite principle of "searching out" the incipient case, and (whether in its seven hundred municipal hospitals or in its growing out-patient and domiciliary practice) treating its yearly hundred thousand or more of patients from the standpoint not of relief, but of prevention—prevention of the occurrence of disease, prevention of the continuance of disease, prevention of the recurrence of disease either in the same or some other person, and prevention of the communication of disease to others, whether inside the family or outside.

Thirdly, that, notwithstanding the ubiquitous existence of these two rate-maintained medical services and public hospitals—notwithstanding also the co-existence of more private medical practitioners than ever, and of a bewildering host of medical charities in the form of voluntary hospitals, dispensaries, and convalescent homes—an enormous amount of illness goes untreated.

### The Courses Open.

Such being the present position, and the facts being, I believe, beyond denial or dispute, the nation has before it the customary three courses. We may do nothing or next to nothing in the way of reform and "muddle along" as we are, with the duplication and overlapping on the one hand, and the appalling amount of unprevented preventable sickness on the other. This is the policy of the *status quo* with such minor improvements as prove easy of achievement, which naturally commends itself to the innate conservatism of the private medical practitioner, as it does to the average Poor-Law guardian. There is, secondly, the policy of "one authority, and one authority only, for all necessitous persons," which, as Sir Arthur Downes sees, means the merging of all the Public Health medical service, all the municipal hospitals, all the health visiting, all the lunatic asylums, all the pensions of the aged, and all the medical and "children's care" work of the education committees in one gigantic Poor-Law service, managed by an enlarged Board of Guardians. I do not imagine that any statesman will think this possible. Finally, there is the policy of breaking up the Poor Law into its constituent services, and transferring each of these services to the rival department that is *actually already in existence* for dealing with that particular section of the population—all the children for whom any sort of public provision has to be made to the Local Education Authority; all the infants, the sick and the infirm to the Local Health Authority; the mentally defective to the Local Lunacy Authority; the healthy aged to the Local Pensions Authority—all these "authorities" being only the Town or County Council acting through different committees—and the able-bodied unemployed to the new National Authority for Unemployment, of which we have the beginning in the National Labour Exchange. These authorities, unlike a Poor-Law system, have as their primary aim not the relief but the prevention of destitution, and, here as elsewhere in life, prevention is not only better but is also cheaper than cure.

### The Minority Proposals.

Now, I do not think that it needs much gift of prophecy to see that, sooner or later, it is this last policy, which is that of the Minority Report of the Poor Law Commission, that is destined to prevail. It may be, of course, too much to expect public opinion generally or the medical profession, the Local Government Board, or the Cabinet, to accept, all at once, any logical or complete scheme. But great is the compulsive power of facts; greater still that of ideas. To take only the question of sickness, and the enormous amount of public money that we have to spend upon it. The evils and the waste of the overlapping and duplication between our two rival rate-maintained medical services are so gross that it is inevitable that we shall, sooner or later, make up our minds to have a unified medical service, in which will be merged both the Poor-Law and the Public Health medical services; both the Poor-Law infirmaries and workhouse sick-wards on the one hand, and the municipal hospitals and phthisis sanatoria on the other. The question is, How will the coming of this Unified County Medical Service affect the voluntary hospital?

### Statistics.

First, let us get the matter into due proportion. No systematic statistics seem to exist, and I am greatly daring to give any figures at all. But just to get some duly proportionate impression of our national hospital service, let me say that it seems that we have in the United Kingdom to-day something like 175,000 hospital beds available for the sick of any kind, in all sorts of institutions. Out of these the separate Poor-Law infirmaries, that represent the last word of institutional perfection in the Poor-Law world, may have some 40,000; the workhouses of the urban districts about 60,000; and the workhouses of the purely rural districts about 10,000—making a total of 110,000 beds occupied by patients actually under medical treatment in Poor-Law institutions (excluding merely senile or imbecile cases). The 700 municipal hospitals of the Public Health medical service have about 25,000 sick-beds, besides some 6,000 more in the corresponding institutions of the Metropolitan Asylums Board, which is essentially a public health authority.

Thus, something like 141,000 hospital beds, or six-sevenths of the whole, are already provided out of the rates and taxes. Then we have the several hundred endowed and voluntary hospitals, providing in the aggregate about 25,000 sick-beds, or one-seventh of the whole. It is a curious fact that the several hundred of endowed and voluntary hospitals seem to be concentrated in not more than seventy towns. There are in the United Kingdom between three and four hundred towns having over 10,000 population, so that it seems that of such towns four out of five (besides nearly all the countryside) are without any sort of voluntary or endowed hospital, and are dependent entirely on one or other form of rate-maintained hospital for every kind of institutional treatment. Voluntary hospitals obviously cannot grow to seven times their present size in order to take over the hospital work of the Poor-Law and Public Health medical services. It is even out of the question to look to the voluntary or endowed hospital for any such increase as would enable it to provide for the 250 or more towns over 10,000 population in which there is no voluntary hospital of any kind, to the same extent as it does for the seventy towns in which such an institution exists. There is no chance for its being able to provide what is requisite for the rural districts. I do not know whether the most enthusiastic "voluntaryist" expects to see the voluntary hospital provide the beds admittedly necessary for the enormous number of patients suffering from phthisis and other forms of tuberculosis, from bronchial and rheumatic affections, from venereal diseases, from such infectious and at present largely neglected matters as measles and whooping cough, and from all the other diseases that now go untreated, or untreated until an advanced stage of disease is reached.

### The Future Local Health Authority.

It is clear that the Local Health Authority of the future—the county or borough council—which will take over and administer the existing public hospitals of the Poor-Law and Public Health medical services, ought to regard these, in the first place, merely as supplementary to whatever voluntary or endowed hospital provision there may be within its area. What will be necessary in each case will be a survey of the available local institutional provision, in comparison with the localities to be served, the number of patients to be accommodated, and the different kinds of diseases and accidents to be dealt with. Such a survey would everywhere reveal deficiencies—often there would be no hospital of any kind available for a particular locality; generally there would be found no institutional provision at all for many diseases; practically nowhere—not even in London, Edinburgh, or Dublin—would the existing institutional accommodation (counting every kind of hospital) be found adequate for all parts of the district and for all sorts of diseases. The first duty of the Local Health Authority, and one that will probably occupy it for many years, will plainly be to supply these manifold deficiencies. In order to save expense to the ratepayers it will only be too eager to make use of every voluntary or endowed hospital, if its governing body is willing, on practically whatever terms the governing body may choose to make. Any involuntary "nationalisation" or "municipalisation" of the existing voluntary or endowed hospitals is, therefore, a question so remote from practical politics that we need not discuss it. On the other hand, if any voluntary hospital, tired of its financial difficulties, preferred to hand itself over to municipal management, it would find no difficulty in the transfer.

### The Status of Hospitals.

What is almost certain to happen, as the Local Health Authority becomes more and more conscious of its responsibility for the health of the community, and as its Unified County Medical Service gets to work, is the conclusion of a concordat with the voluntary hospital as to the cases that it desires to receive and the terms on which it will treat patients sent in by the public authority. Do you desire to have cancer cases, accident cases, phthisis or childbirth, the common ailments or "interesting" cases? The Local Health Committee and the Unified County Medical Service will gladly select from among its patients needing institutional treatment whichever classes the voluntary or endowed hospitals of the district are willing to receive—in the first place because this will ease the pressure on the Local Health Authority to provide additional institutional accommodation at the cost of the rates; and, in the second place, because the more specialised voluntary or endowed hospitals, with their superb visiting staffs, will be able to afford the best possible treatment of these selected cases. These hospitals will, therefore, be assured of a constant and exactly graduated supply of exactly the kind of cases that they may desire to deal with.

If these hospitals are content, as they often are at present, to receive these cases without payment, the Local Health Authority would have no ground whatever for claiming to intervene in the administration in any way. If, on the other hand, the hospitals choose to ask for any contribution out of public funds, in part return for the valuable public services that they would be performing, the Local Health Authority would naturally expect, as in all other public services, in the first place to satisfy itself by occasional expert inspection that the work was being properly done; and, in the second place, to nominate a limited number of public representatives to sit on the hospital governing bodies. It would be for these governing bodies to decide whether they preferred to go on without contributions from the rates, and without public representatives on their governing bodies, or to accept contributions from the rates, at the price of having to admit public representatives to these governing bodies. That issue, on which I believe much difference of opinion exists, is not affected by the proposed merging of the Poor-Law and Public Health medical service into a Unified County Medical Service.

### Reforming the Out-patient Department.

The public advantage of absolutely gratuitous and unrestricted access to a mere general out-patients' department of a general hospital—where no study of the domiciliary conditions, no "interrogation of the functions," no consideration of the diathesis, and no sort of disciplinary supervision of the patient is possible—seems to me, as a lay person, at least open to question. What would be the advantage to the hospital itself of such a general out-patients' department—what real credit it would bring to any voluntary hospital—I am not able to estimate. From all that I have been able to learn of these general out-patients' departments of the general hospitals, large or small, I confess that I should imagine that the hospitals would, for the credit of twentieth-century medical practice, really be glad to be able to relinquish them. The case is, of course, different when the out-patients' department is specialised, and amounts, more or less, to the consultation of a specialist, on the advice of the patient's own doctor.

### The Financial Question.

There remains one question—perhaps the most vital of all. What effect will the coming of the Unified County Medical Service be likely to have on the subscriptions and donations, and on the legacies, made to voluntary hospitals? At first sight I am tempted to reply, none whatever. Why should it make any difference to the potential donor or testator whether the Poor-Law and Public Health medical services exist in their present overlapping and duplication, or whether they are merged in a Unified County Medical Service? What may make a difference to him is, not the form of this County Medical Service, or whether it exists in one body or in two, but whether or not the Public Authority is believed to be adequately carrying out what are actually its existing duties under the present Public Health Acts, in providing out of the rates whatever hospital accommodation the district requires. This, however, does not depend on the coming of a Unified County Medical Service.

If a county or borough Council (like the Widnes Town Council or the Barry Urban District Council at the present time), finding the district unsupplied with hospital accommodation, sets up a hospital out of the rates for non-infectious cases, it undoubtedly will deter the potential donor or testator from setting up another. The 700 municipal hospitals that have already been provided out of the rates, primarily for infectious cases, have no doubt diverted to other purposes some charitable donations that would have been given, here and there, to meet the need that would otherwise have been felt. But because the town councils have provided hospitals for this and that purpose, and will go on providing more and more, it does not follow that this prevents any private donations to hospital maintenance generally. On the contrary, we have witnessed, in the course of this generation, alongside of a municipal expenditure on hospitals in capital outlay of probably four or five millions sterling in the last thirty years, an expenditure out of voluntary contributions probably amounting to something like that sum. The truth is that this is a field in which appetite grows with eating. Let the municipality do what it will, there will always be people anxious to contribute to hospitals; there will be always even an incessant growth of new and additional hospitals, specialist hospitals, and specialised out-patients' departments, hospitals to carry out this or that new treatment, local hospitals, sanatoria for phthisis, convalescent homes for all kinds of patients, hospitals for special classes or particular races, hospitals relying on this or that kind of management, this or that kind of patient, this or that kind of medical staff.

To me, at least, it seems that the field for voluntary hospitals will even be enlarged as we learn more and more what is needed. And it is good that this should be the case. The more voluntary effort and personal philanthropy can be induced to supplement the indispensable operations of the Local Health Authority the better. What is wrong is only the desire of some people to get rid of the Local Health Authority altogether, and to do its work themselves.

---

### Discussion.

Mr. James R. Motion, Chief Inspector, Glasgow Parish Council, dealt fully with the various points raised in the speech, and in the course of his remarks said: As to the policy of breaking up the Poor Law into its constituent services, and transferring each of these services to the rival department that is actually already in existence for dealing with that particular section of the population—children to the education authority; infants, the sick, etc., to the

Public Health Department, and so on—is it seriously meant that the home is to be invaded by five separate parties and their officials? Are the searchers to ferret out once a week, month, or year, and is the sanctity of the home to be liable to invasion any moment? I am almost certain that if the unified County or City Medical Service comes, then what with this and the other claims upon the rich and middle class alike by the Central and Local Government authorities, there will be a noticeable shrinkage of contributions from the charitable public. On the other hand, it has long been felt as a striking grievance that patients who could easily afford the total or partial cost of their treatment in the infirmaries of the country are maintained at the cost of the institution, and it seems to me that if some regulations were applied in such cases it would have the effect of having many more beds available for the poorer classes. There are hospitals of all kinds, sufficient, I think, for the needs of the public, if some process of selection was adopted, and although some of the institutions may not be judiciously placed as regards population, etc., that is not nowadays, with motor and other ambulances, a serious difficulty. Our experience in Glasgow is that about 25 per cent. of the applications for parish relief are not genuine cases, and that they might have kept off the parish with reasonable care. It is the experience of nearly all charitable societies as well that many recipients endeavour by fair means or foul to get aid from different sources, although this is largely being remedied through the central register kept by the Charity Organisation Society. It is well known that the friendly societies of the country afford great and beneficent relief to many thousands of members, while only a very small proportion require medical assistance of any kind. I am afraid I have taken up too much of your time, but in conclusion let me say in justification of my opinion that Mrs. Webb's paper is the result of her experience in England and not of Scotland; that she was good enough to indicate to me many of the points which afterwards appeared in the Minority Report of the Commission at a time when the oral evidence in Scotland had only partially been taken, and I think I have indicated in a general way that the Poor Law administration of the two countries is essentially different in principle. Reference was made the other day with regard to the medical provision for citizens. That word struck me. The great bulk of the people who come to the Poor Law service are not citizens—never have been and do not intend to be—and, necessarily, some organisation, such as the existing Poor Law, must deal with those cases which come from all quarters of the country bare-faced, telling us that the new hospitals of the Glasgow parish are far preferable to anything in the country. Dr. Chalmers mentioned yesterday that there were too many dispensaries, and I entirely corroborate that. I have seen the result of it. It is not the first time that I have been in a poor person's house where the woman had three separate bottles on the mantelpiece got from three separate dispensaries.

### The Voluntary System and Abuse.

Councillor John Macpherson (Edinburgh): I have heard a great deal about hospitals of various kinds, but I suppose we are pretty well agreed that the greatest abuses are to be found with our voluntary hospital system. First of all let me say that I consider the voluntary hospital one of the grandest institutions of our country, whose objects are to bring to the aid of the poor and needy sufferer that help and assistance, medical and otherwise, which they are not in the position to get for themselves. The abuse, in my opinion, consists in this, that a great many well-to-do and selfish citizens who are well able to pay for their own medical treatment and hospital assistance if necessary do not do so, but come and take advantage of the contributions of the charitable public and the charitable medical profession. The results are, first of all from an administrative point of view, to put an intolerable strain upon the management of the hospital, and to displace by their admission the people for whom the hospital was originally intended and for whose assistance the generous contributors desire to find a place. I think that in a great representative gathering like this it should go out with no uncertain sound to that selfish section of the well-to-do community that the hospitals are not intended for them, and that it is their duty, instead of going there, to take advantage of the generosity of other people, to contribute themselves towards extending these privileges and blessings to people who are not so able as they are to get the advantage of the hospital.

### Unification.

Dr. Robertson (Medical Officer of Health, Leigh): I should like to make one remark only as regards unification. While it may be absolutely necessary to have a united medical service, the difficulties of administration of such will be enormous. From one point alone, I think, most medical authorities will be in agreement, that while such a disease as tuberculosis is now being looked upon as an infectious disease, it seems to me fairly apparent that such a disease should be administered by the authority which is responsible for the control of infectious diseases, and while the Poor Law authorities have been in the habit of dealing with advanced cases of tuberculosis, local authorities and public health authorities have been taking the early cases under their wing, and there is therefore no reason now why the public health authorities should not take advanced as well as early cases under their control. This would help a great deal to curtail and arrest the spread of this disease, because the local authorities themselves are dealing with the housing conditions of the people and the Poor Law authorities are not, and there is the environment of the people where the two conditions require the most control to arrest this disease. The general subject of unification of hospitals is not one which appeals very much to a medical officer of health, because, as I have already said, he is confining his attention to the control of infectious disease, and if anything is done at all my sympathy would be in the direction of placing tuberculosis under the ægis of the local authorities and unify that part of the system.

### Pay Wards and Paying Patients.

Dr. Nathan Raw: May I say a word upon this important paper which has just been read on behalf of Mrs. Webb? I am glad first of all to learn that Mrs. Webb does not advocate a change in the voluntary system of hospitals, because I think there is an impression abroad amongst the public that the Minority Report advocates the total abolition of the voluntary system, and that the whole of our hospitals should be merged into one municipal authority. Now, from my experience of both voluntary hospitals and the Poor Law system, there can be no doubt that the voluntary system is doing and has done an enormous amount of good both to Scotland and to England, and for this magnificent system to be swept away would be quite unthinkable. First of all, the voluntary hospitals provide the very highest and best skill for the treatment of the poor and those who enter their doors, and again, the education of the future generation of medical men is of such supreme importance that the voluntary system as at present would be the only system which could undertake the education of the medical student. Therefore, for these two reasons I hope that the voluntary system will remain as it is, and I have no fear that it will; but I think that the relation between the present voluntary system and the municipal or Poor Law system, as we may call it, is anomalous, and for this reason. I have heard it said that the system in Scotland is better than in England. I have had the opportunity and the privilege of administering a hospital in Scotland, and also one in England, and I quite admit that the broad principle of the treatment of the sick in Scotland and the hospital system generally is in front of that which is at present in force in England. Now in England, which I have perhaps more right to speak of on the Poor Law system, if a person is taken into a Poor Law infirmary, suffering from any disease whatever, he is called upon to pay as far as his means will permit. Even his furniture and his property may be seized or taken in support of his payment to the hospital. Now if the same person is forced to go into a voluntary hospital, he is not asked for a single penny, and he goes there and receives treatment without any payment whatever. Under the Poor Law system, if a man enters a Poor Law infirmary he loses his municipal and Parliamentary votes. In a voluntary hospital nothing is said, and I think the feeling between the voluntary and the Poor Law hospital would entirely disappear if both classes of people were asked to pay according to their means. I am sure that that is perfectly fair, and that the voluntary hospital should insist on all patients paying according to their means for the value that they get out of the institution. I am sure you will agree that the poor who go into the Poor Law hospitals should not be compelled to pay when those who go into the voluntary hospitals are not asked for a penny, and I am sure it could be arranged between the voluntary hospital and the city authorities and the Poor Law authorities. I am sure that some combination or co-operation could be found between the voluntary hospital and the municipal hospital, and I think the municipal authority would be well advised to subsidise the voluntary hospital in their efforts to treat the sick poor. Although I do not want to see the voluntary hospital municipalised entirely, if a working arrangement could be made by which it could be assisted in financial difficulty, I am sure that the benefit would be all round.

### Mrs. Webb's Mistakes.

Councillor W. F. Anderson (Glasgow) : I am certain that if Mrs. Webb thinks that the municipality will be entitled to rate for the administration of hospitals, and that it will not affect the usefulness of the hospital, she is making a great mistake. Let the public of Glasgow know that the hospitals are there for people who require medical assistance and who are not in a position to pay for it, and anything that we can do to redress the wrongs which undoubtedly exist will be of very great benefit; but as long as there is a tremendous discrepancy among the middle-class people—say, a clerk with £150 a year, who is subject to the same ailments as other people—as long as he is not in the position of paying great fees to medical men and nursing homes, he has only two options—either not to get the operation done so well at home, or go into an hospital where he will get the best skill that science can bring him. I am not advocating sweating at all, but if a man has a limited income and has a very serious and expensive illness, he has only these two options; there is no middle course, and I do suggest that the medical men, for whom I have the most profound reverence because of what they have done and the sacrifices they have made in public health—if they would only think out the problem, I think that would be the best way to relieve our hospitals of a great many people who have no right to be there.

### The Broad Outlook.

Dr. D. J. Mackintosh, M.V.O., Medical Superintendent, Western Infirmary, Glasgow : It was not my intention to make any remarks, but when I was asked to become one of the honorary secretaries of the British Hospitals Association, I understood that the Conference would confine itself to discussing the hospital situation as it at present exists, from a broad standpoint and not from any local standpoint, and deal with hospitals generally, not municipal hospitals, Poor Law or voluntary institutions, but try to grapple with the whole situation, and in that way do some good. I take it that the object of this Association is the discussion of principles, and that local details will be referred to the various committees which will be representative of the various interests, and after these committees have deliberated upon the points raised, the matter will be brought up at the next Conference to be dealt with there. At a recent meeting of the British Medical Association I pointed out that the administration of English and Scottish and Irish hospitals differed materially. Speaking more especially of the Poor Law hospitals, Mr. Motion said yesterday that, as far as Poor Law hospital relief was concerned, Scotland was years ahead of England. What I believe will help us more than anything else at the present time is more direct and active co-operation between our various agencies. There is not the slightest doubt that what is called abuse would not then exist; at least, if it did exist, it would be to a very much less extent; but I suppose, as long as people get anything for nothing, there will always be some people who will take advantage of it. As far as abuse is concerned, I do not know that it is quite the correct term to use in this connection. I have been twenty-six years resident in Glasgow hospitals, and my experience has been varied. I have been in an eye infirmary, a fever hospital, and two general hospitals. I have been for nearly nineteen years in the Western Infirmary, and I come into daily contact with people who are applicants for admission. I object to those people who apply for treatment being regarded as abusing the hospitals as the term is generally understood, so long as you make no other provision for them and they are recomended by their own medical attendant. There is practically no provision in Glasgow for the upper tradesman or clerk or the man in the warehouse, and surely you do not say that he is abusing the hospital when he has no other method of getting what he requires. He or one of his family may have to undergo a serious operation, and the surgeon won't do it at home; and even if he did, the surroundings are not such as would tend to recovery. I object to treatment in a hospital being called an abuse so long as there is no other provision being made for this class. I am glad to find in Mr. Finnie's paper that he referred to out-patients alone when he spoke of abuse. I am referring to the in-patients; that is where the money is spent.

### Payment of Hospital Staffs.

Just a word regarding the medical and surgical staff of our hospitals. I believe that every professional man should be paid adequately for his services either in a hospital or elsewhere. I have the honour to belong to the medical profession, and no one would guard its interests more jealously than I would, but I am afraid that we must look at this subject from a broader point of view if we are to draw any general conclusions. During the last eighteen years I have observed that the number of hospital students has fluctuated from time to time. I have asked business men and professional men and men for whose opinion I have the utmost respect if they could account for this. It has been suggested that in the years when trade is bad—perhaps general business, or the woollen business, or manufacturing business of any kind—a man says that he will not put all his boys into his own business, and he will put two of them into a profession. The medical profession is frequently chosen; at any rate, these young men are put into a profession, irrespective of whether the profession at the time requires to be increased or not. I believe that it is accepted in the general sense that when trade is bad there are more men entering the medical profession at that time than at other times. If we are going to arrive at any satisfactory conclusion as to how the poor, the middle-class, and the rich alike are to be treated—because a sick man is a sick man whether he is rich or poor—we cannot look at it entirely from the point of view of the medical profession, and we must not view the problem too narrowly. We must take it from a broad standpoint and say, "Who sent these people to the hospital?" Now, as a superintendent, I am prepared to say that the bulk of the cases that at first sight one might regard as cases unsuitable for hospital treatment are sent direct by the medical men themselves, and I do not blame them. I say that the medical man who is attending the wife of a tradesman or a clerk is in a better position to judge as to whether that patient should go into a hospital and be treated there than any critic outside. If he sends that patient he knows that the patient is not able to pay him and cannot possibly get the necessary treatment outside. Therefore they come to a general hospital. Is that abuse? Abuse is hardly the proper term until we make some provision for these middle-class people having treatment in the same way as the poor get and the rich pay for. As the result of this Conference, if the Poor Law and municipal authorities and the directors of voluntary effort, of whatever kind they may be, either in general hospitals or sanatoria or special hospitals, would co-operate more directly, we would have less overlapping. A great deal could be done by co-operation, the sick would be better treated, and there would be less outcry about abuse.

### What is Abuse?

Mr. Johnson (Sick Children's Hospital, Great Ormond Street, London): I cannot help feeling that this controversy about hospital abuse has gone on for a long time, because we do not define what we mean by abuse, and we always mean different things. There are two kinds of hospital abuse. You may mean, first of all, that the hospital is drawing too fine a line—that is to say, that the hospital and the person whom you accuse of abuse fix a different limit; and the second kind of abuse is the hospital being deceived as it has set up its own standard, and allowing people to come in who are above that standard. These two kinds of abuse seem to me totally different things, and have to be dealt with in a totally different way. Taking the first kind, where do you draw the line? It is extremely difficult to say, because you cannot take a wage limit. If you take two people with 30*s.* a week, one may be comparatively poor and the other comparatively rich. It is not what a man has, but what he has in relation to his needs. I think we can get at it in another way. There has been a great deal of research lately into the question of poverty, and we have Rowntree's report, which shows that in a large town and a small town the amount of poverty is about equal, affecting about one-third of the inhabitants, and I think that we are laying ourselves out to treat one-third or one-half of the population. Now, as regards the second kind of abuse; that is precisely the same problem which occurs in many walks of life. When one comes to study the question very attentively, what strikes one is that the people who come most nearly to abuse are the people who are perhaps just on the borderland of being admitted to Poor Law relief. These are the people who get the most benefit from the hospital. For the very poor I am inclined to think that one wants something more than the voluntary system, although I think the voluntary system works very well indeed. I quite agree with what Dr. Mackintosh says about co-operation with other authorities so as to attack the problem from all sides—not from the medical side, but from every side—and in the meantime we can only go on and see that at any rate no physical infirmity is likely to be the cause of people being poor, and one of the most efficient means in that direction is conferences like this where we can discuss the means of bringing about greater co-operation between all the authorities.

### The Value of Discussion.

Dr. John Glaister (Professor of Forensic Medicine and Public Health in the University of Glasgow): If the controversy on the Poor Law Report from the Minority side does anything at all beneficial, it does this great thing; it directs the attention of the public to what they have hitherto too long neglected. Now, what is the crux of the controversy? It seems to me to be this. There are three courses open to us—to leave things as they are, and that is the easiest thing to do; the second is to take the present system and try to improve it. These two may be classified as one. Taking my second point as being an example of the present type; the present system improved, you have a large authority corresponding to the Poor Law authority which will deal with the whole system of medical service throughout the country and embraces within it everything—public health service, Poor Law service, and voluntary hospital service; it forms a gigantic clearing-house by which patients will be sorted out and sent to different establishments with no overlapping and no trouble. That is an ideal system on paper, but I have yet to learn from my knowledge of human nature that it is at all workable. The third system is the destructive policy with regard to one agency, namely, to break up the Poor Law system as it stands and distribute the different branches of the Poor Law through what is called agencies already in existence. Now, if you look carefully at Mrs. Webb's paper you will see the agencies which are supposed to be in existence, and under cover of the Poor Law authority you will find that the children for whom any sort of public provision has to be made are to be turned over to the local education authority, because they have school officers and arrangements for feeding them and, I suppose, clothing them and all the rest of it, and then the infants, the sick, and the infirm are to be turned over to the local health officer. Obviously you will require to municipalise your hospitals and provide an infant hospital and a hospital corresponding to our voluntary hospital for the ordinary sick and the infirm, because they cannot house these people in the present infectious hospitals.

### Self-inflicted Ills.

Then you have to deal with the thorny question of the illnesses which people bring on themselves. In our tendency to do good to human beings in these days I think we are forgetting one sanitary principle. We are forgetting to denounce the person who brings upon himself his own evils. We are forgetting the sanitary principle of St. Paul, and I would extend that principle further; that if a man will not work, neither shall he eat; and I think the more we press home that principle the better. A man who brings disease upon himself is a public nuisance and a public danger, and he has to be dealt with. Now, while I have said that there are three courses, it seems to me that in any State where, like Topsy, things have grown, it will be impossible to change those which have grown up in a century and a half principally in hospitals, or it will be impossible to change them sufficiently, because you will be unable to convince people that things will be better done. In the first place, most people are agreed that while the voluntary system has been a characteristic of Christianity throughout the world, it has done most excellent service when the State never thought of doing anything for anybody. After Christianity came the Poor Law principle, and then by the progression of the law came next the public health infectious hospital. I would be sorry that the day should ever arrive when the voluntary system should be put down or discouraged, and I am entirely at one with Bailie Anderson that as soon as you municipalise the hospital you kill the man who gives donations, and the post-mortem benefactor will not exist. People are averse to being coerced into paying rates and at the same time give a donation. I am not here to say that the present system cannot be improved. My experience is that it can be improved, but it can only be improved slowly. The first, I think, is in the direction indicated that there should be a closer co-operation between the different branches of the public medical service. That is gradually growing, and growing of necessity.

### Hospitals and Consumptive Patients.

The great problem of the voluntary system is this, what are we to do with the people who are to be dealt with in the next paper? There is no trouble nowadays if municipal authorities like to put their powers into operation to deal with the ordinary pulmonary cases, because by order of the Local Government Board municipalities are now empowered to put tuberculosis and phthisis amongst the notifiable diseases, and therefore the law falls in, but the question of the voluntary system is, what are we to do with our tuberculosed joints or spines? I am here to say that some of these cases take up a bed for a whole year at a time. That is a very serious problem for the voluntary hospital. There is a gap here that has to be filled up in another way, but this is not the time to discuss that; and let me say that it will be a godsend to this country, whether the provisions of the Minority Report are carried out or not, when the question has been ventilated in such meetings as this and in such papers as those that we have heard.

